# Effect of Eutectic Silicon on the Electrical Conductivity of Al-Si Alloys Using Principal Component Regression Analysis

**DOI:** 10.3390/ma19122591

**Published:** 2026-06-16

**Authors:** Bin Li, Zhao Yang, Yifan Li, Jianqi Lu, Lijia Tan, Wenhao Gong, Qinghuan Huo

**Affiliations:** School of Materials Science and Engineering, Central South University, Changsha 410083, China; libin0711@csu.edu.cn (B.L.); lyf@csu.edu.cn (Y.L.); 223102041@csu.edu.cn (J.L.); believer021@163.com (L.T.); 18296952922@163.com (W.G.); huoqinghuan@csu.edu.cn (Q.H.)

**Keywords:** Al-Si alloys, eutectic silicon, electrical conductivity, principal component regression, electron scattering

## Abstract

The microstructure of as-cast Al-xSi (x = 4, 7, 10) alloys solidified under various cooling rates was characterized using scanning electron microscopy (SEM). To overcome the multicollinearity among eutectic silicon parameters, Principal Component Regression (PCR) analysis was employed to quantitatively evaluate the effects of silicon morphology, scale, and content on the electrical conductivity of the alloys. The results demonstrate that rapid solidification significantly refines the plate-like eutectic silicon and reduces its volume fraction, leading to improved electrical conductivity. The PCR model shows that a hierarchical mechanism: volume fraction (PC1) acts as the principal determinant, increasing baseline resistance primarily by truncating the electron mean free path (MFP); meanwhile, within identical alloy systems, morphological parameters (PC2) play a dominant regulatory role. A semi-quantitative electron drift path model was established, confirming that the morphological deviation of eutectic silicon from a spherical shape (i.e., increased aspect ratio) causes a non-linear increase in the amplitude of electron detours. This geometric elongation significantly degrades electrical conductivity, providing theoretical guidance for the microstructural design of high-conductivity Al-Si alloys, which can be practically applied to the manufacturing and optimization of lightweight, heat-dissipating enclosures for new energy vehicle (NEV) motors and power distribution systems.

## 1. Introduction

Owing to their excellent castability, high specific strength, and strong electrical and thermal conductivities, Al-Si alloys are widely utilized across the automotive and 5G communication sectors. The current boom in new energy vehicles (NEVs) has brought about much stricter standards for both vehicle lightweighting and overall electrification. In practice, utilizing cast aluminum significantly reduces the weight of NEV electric motors and power distribution systems. At the same time, the inherent corrosion resistance and heat-dissipation capabilities of these alloys naturally extend the working life of the internal electrical components. Because they successfully balance these mechanical and thermal demands, Al-Si alloys have emerged as the premier choice for manufacturing the protective enclosures of NEV motors and power systems [1,2,3].

Generally, the physical properties of Al-Si alloys are highly dependent on the micro-morphology of eutectic silicon. Under conventional solidification conditions, coarse, plate-like eutectic silicon not only easily induces stress concentration—acting as a source for crack propagation and reducing the alloy’s elongation—but also significantly impairs its electrical conductivity [4,5,6,7]. By adding Sr modifiers and increasing the cooling rate, the primary α-Al grains can be refined and the size of eutectic silicon reduced, thereby simultaneously improving the material’s strength, plasticity, and electrical conductivity [8,9,10,11]. For example, studies by Wang et al. [12] and Vandersluis et al. [13] demonstrate that refined silicon morphology enhances the electron mean free path. A critical evaluation of the existing literature reveals a specific research gap: empirical studies document macroscopic conductivity improvements but fail to decouple the interacting microstructural variables. Additionally, classical theoretical models for bulk conductivity (e.g., Maxwell-Eucken) assume idealized spherical inclusions. By neglecting the complex geometric tortuosity of actual three-dimensional eutectic networks, these existing models cannot accurately calculate the electron-scattering cross-section defined by the specific morphology of the Si phase [14,15,16].

Previous studies have focused on the refinement of eutectic silicon by increasing the cooling rate or adding densifiers, and although the increase in conductivity has been observed macroscopically, a regression model for the quantitative relationship between the geometrical characteristics of eutectic silicon (size, distribution, and morphology) and conductivity is lacking [17,18]. The core difficulty lies in the severe multicollinearity among the statistical indicators of eutectic silicon, which causes traditional linear regression to fail. Motivated by the material requirements for NEV protective enclosures, this study aims to resolve this mathematical coupling and establish a quantitative predictive model. To achieve this, high-purity raw materials and rapid melting were used to exclude the influence of trace elements. A custom-designed mold was employed to achieve varying cooling rates for the same alloy composition within a single casting cavity. By combining quantitative microstructural analysis via Image-Pro Plus 6.0 with Principal Component Regression (PCR) using SIMCA 16 software, this study aims to eliminate covariance interference and construct a physically meaningful conductivity prediction model, providing a basis for the design of high-performance aluminum alloys.

## 2. Materials and Methods

**Figure 1:**
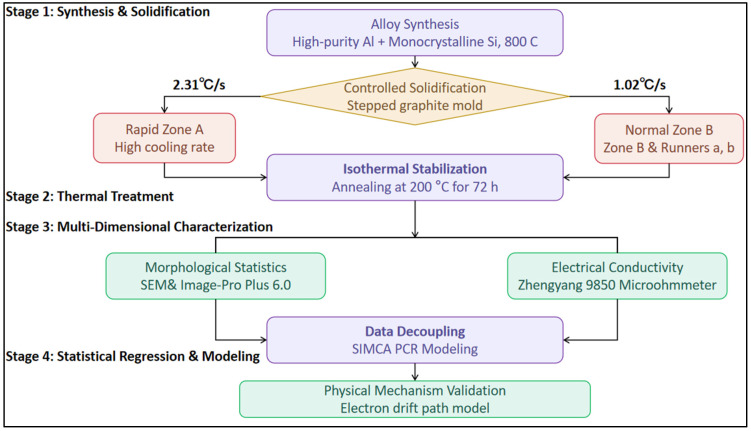
Schematic flowchart of the overall experimental workflow.

The experimental design of this study systematically connects alloy synthesis, macro-gradient solidification, stabilization heat treatment, and subsequent quantitative characterization. To clarify the sequential logic and execution paths of the methodology, the complete experimental workflow is schematically outlined in Figure 1.

**Figure 2:**
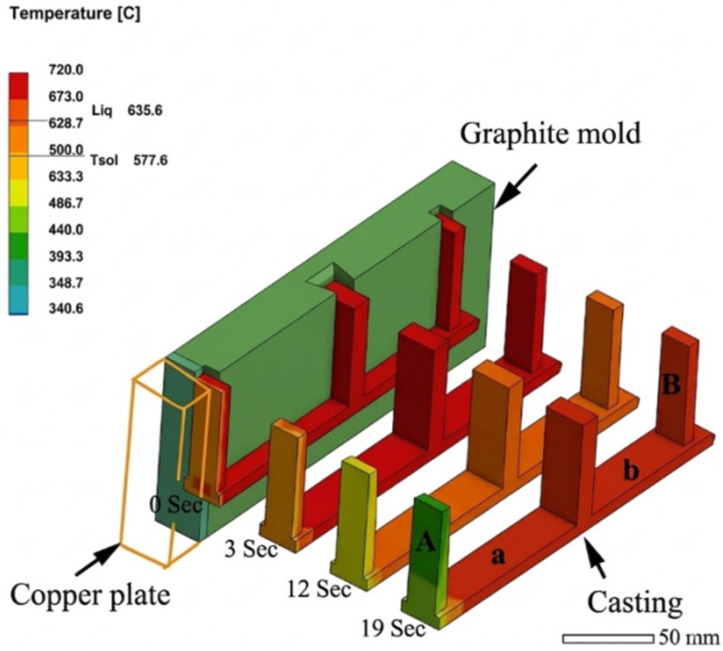
Diagram showing the variation in the temperature field in Al-Si castings.

High-purity aluminum ingots (99.995 wt.%) were melted at 800 °C in a medium-frequency induction furnace using a high-purity graphite crucible. Subsequently, high-purity monocrystalline silicon (99.9999 wt.%) was added, and the melt was held at 800 °C for 5 min to ensure uniform mixing. The alloy was then cooled to 720 °C and poured into a stepped high-purity graphite mold, which had been preheated and held at 400 °C for 1 h in a KSL-1100X muffle furnace (Hefei Kejing Materials Technology Co., Ltd., Hefei, China). As shown in Figure 2, the stepped casting cavity features a rapid-solidification zone (Zone A), a normal-solidification zone (Zone B), and two inner runners (Channels a and b). A room-temperature copper block was integrated into the outer wall of the rapid solidification zone to accelerate the cooling rate. Figure 2 presents the simulated temperature fields at 0, 3, 12, and 19 s after filling, obtained using ProCAST 2021 software. It is evident that the copper block exerts a significant cooling effect on the molten aluminum. In order to exclude the interference of non-equilibrium solid solution atoms on the lattice distortion and to focus on the observation of the effect of eutectic Si morphology on electron transport, all cast specimens were subjected to an isothermal stabilization treatment at 200 °C for 72 h to promote the full precipitation of solid solution atoms and eliminate the effect of non-equilibrium eutectic and solid solution Si [19,20,21].

The samples were mechanically polished and subsequently electropolished (using a solution of perchloric acid and ethanol in a 1:9 volume ratio) to observe the three-dimensional morphology of the eutectic silicon by Quanta-200 scanning electron microscope (FEI, Hillsboro, OR, USA), operated at an accelerating voltage of 20 kV with a probe current of 1.0 nA, a spot size of 3.0, and an aperture of 30 μm. More than 10 photographs were taken for each sample at 500× and 1000× field of view, and the area, aspect ratio, and surface density of the eutectic silicon were quantified according to the principle of stereology. The quantitative statistics of eutectic silicon is performed by Image-Pro-Plus 6.0 image (Media Cybernetics, Rockville, MD, USA) analysis software, and Roundness is introduced to characterize its geometrical regularity [22]:

(1) $$S\;=\frac{4\pi A}{P^{2}},$$

Roundness (*S*) is defined based on the area (*A*) and perimeter (*P)* of the particle, where the closer *S* is to 1 indicates a greater tendency toward a sphere, and vice versa, a greater tendency toward a more complex or sharp shape.

The resistivity at room temperature was determined using a Wheatstone bridge, and the equipment used was a Zhengyang 9850 microohmmeter (Zhengyang Instrument Co., Ltd., Changzhou, China) with an accuracy of 0.01 microohms. The measurement temperature was 23 ± 2 °C. The statistical software was SIMCA 16 (Sartorius Stedim Data Analytics AB/Umetrics, Umeå, Sweden).

## 3. Results

**Figure 3:**
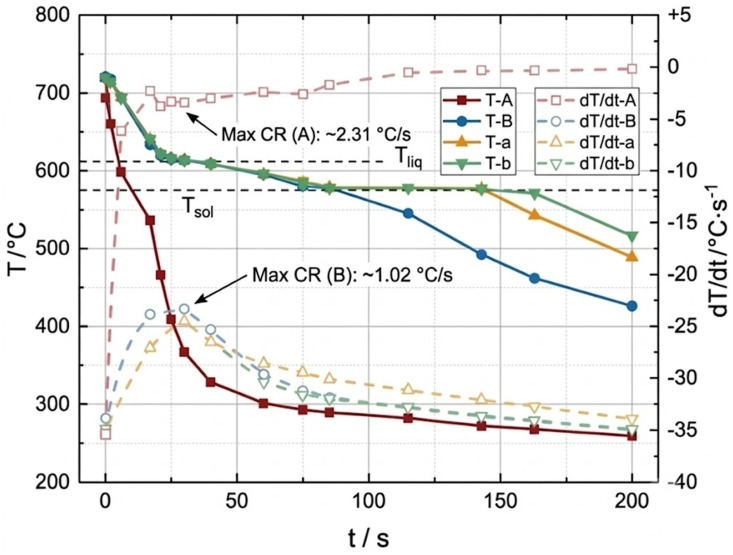
Casting temperature curves with time. Solid lines represent the simulated temperature profiles, and dashed lines indicate the corresponding cooling rate curves for the Al-7Si alloy at the rapid solidification zone (A), normal solidification zone (B), and inner runners (a, b).

Figure 3 (solid lines) illustrates the simulated temperature profiles obtained via ProCAST at specific reference points: 50 mm above the casting bottom in both the rapid and normal solidification zones, and 3 mm above the bottom at the inner runners. The data reveal that the rapid solidification zone experiences a maximum cooling rate of 2.31 °C/s. In contrast, during the initial solidification stage, both the normal solidification zone and the runners exhibit an identical cooling rate of 1.02 °C/s; during the subsequent cooling phase, the cooling process in the normal zone accelerates significantly, maintaining a cooling rate nearly 1.4 times that of the adjacent runners. The Al-7Si alloy was selected as the representative case because its eutectic silicon content lies in the intermediate range among the three compositions studied (Al-4Si, Al-7Si, and Al-10Si), making it most representative of the morphological transitions observed across all alloys under varying cooling rates. Using the Al-7Si alloy as a representative case, the temperature curves for the rapid solidification zone (Location A), the normal solidification zone (Location B), and the two runners (Locations a and b) were differentiated with respect to time. The resulting cooling rate curves are plotted as dashed lines in Figure 3.

### 3.1. Eutectic Silicon Morphology and Limitations of Traditional Multiple Linear Regression

**Figure 4:**
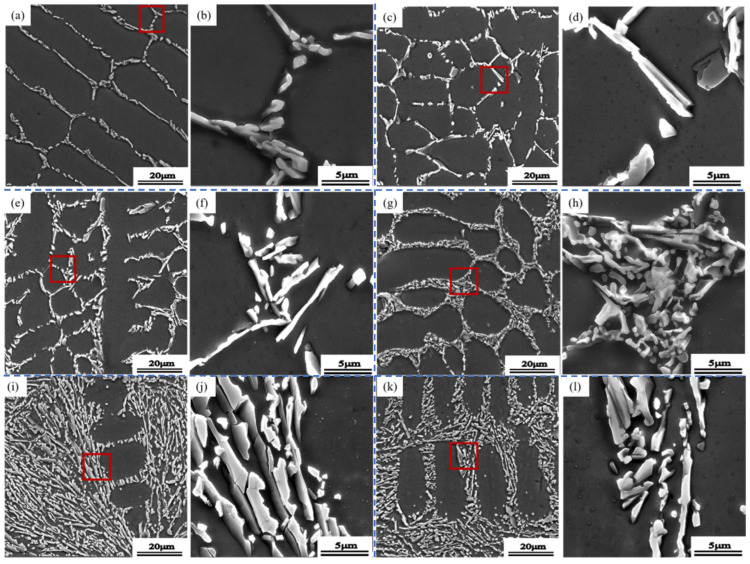
Morphology of eutectic silicon by SEM: (**a**) Dendrite morphology of Al-4Si-1-A; (**c**) Dendrite morphology of Al-4Si-1-B; (**e**) Dendrite morphology of Al-7Si-1-A; (**g**) Dendrite morphology of Al-7Si-1-B; (**i**) Dendrite morphology of Al-10Si-1-A; (**k**) Dendrite morphology of Al-10Si-1-B* (**b**,**d**,**f**,**h**,**j**,**l**) are corresponding enlarged images with red frame.

Figure 4 presents the secondary electron (SE) images detailing the three-dimensional morphology of the eutectic silicon across the three alloys. In these micrographs, the white regions correspond to the Si phase, while the dark regions represent the α-Al matrix. As illustrated, the Al-Si alloy exhibits a typical irregular eutectic microstructure. Coarse, lath-like eutectic silicon particles interlock within the three-dimensional space, deeply embedded within the continuous aluminum matrix. This morphological evolution is governed by the fundamentally distinct growth mechanisms of the two phases. During solidification, the non-faceted α-Al phase tends to grow isotropically. Conversely, the highly faceted eutectic silicon is strictly constrained by crystallographic anisotropy, extending preferentially along specific <110> crystallographic directions to form lath-like structures [18].

To establish a quantitative mathematical correlation between this complex morphological evolution and the macroscopic electrical conductivity, comprehensive statistical analyses of the geometric features and spatial distribution of the eutectic silicon were conducted using Image-Pro-Plus 6.0 software. Six core parameters were extracted to thoroughly characterize the eutectic silicon. Parameters describing the morphology of individual silicon particles include the average area (X_1_), aspect ratio (X_2_), and roundness (X_3_). Furthermore, parameters reflecting the collective spatial distribution and overall content comprise the volume fraction (X_4_), surface density (X_5_), and the actual mass fraction of added silicon in the alloy (X_6_).

Correlation of the quantitative eutectic silicon data with the electrical conductivity measurements reveals that the influence of eutectic silicon on conductivity is not dictated by any single morphological factor. Consequently, multiple linear regression (MLR) was employed in this study to elucidate the impact of the individual statistical parameters on the electrical conductivity of the alloy. MLR is a well-established statistical method for evaluating how a set of independent variables simultaneously affects a single dependent variable. Assuming a system with k independent variables, the general formulation of the regression model can be expressed as

(2) $$Y={\;\beta }_{0}+\beta_{1}X_{1}+{\;\beta }_{2}X_{2}+\ldots +{\;\beta }_{K}X_{K}+\varepsilon ,$$

where $\beta_{0}$ is the regression constant (intercept), $\beta_{1},\beta_{2}\ldots \beta_{K}$ represent the partial regression coefficients, $X_{1},X_{2}\ldots X_{K}$ denote the independent variables, and ε is the residual error term. In the present regression analysis evaluating the effect of as-cast eutectic silicon on electrical conductivity, the alloy’s conductivity was defined as the dependent variable (Y). Concurrently, the morphological and distributional parameters of the eutectic silicon—namely, area (X_1_), aspect ratio (X_2_), roundness (X_3_), volume fraction (X_4_), surface density (X_5_), and mass fraction (X_6_)—were selected as the independent variables. The raw statistical data are summarized in Table 1. Based on these data, multiple linear regression was executed using Origin 2024 (OriginLab Corporation, Northampton, MA, USA) software, yielding the following empirical model:

(3) $$Y\;=\;-0.05X_{1}\;-\;4.54X_{2\;}-\;0.35X_{3}\;+\;3.37X_{4}-\;21.34X_{5\;}-\;83.57X_{6}\;+\;55.61,$$

Preliminary analysis of the regression model indicates that the area, aspect ratio, volume fraction, surface density, and mass fraction of the eutectic silicon all exert a negative influence on electrical conductivity, which aligns with the theoretical expectation that all structural parameters of eutectic silicon (larger size, elongated morphology, higher volume fraction, and greater content) impede electron transport and thus reduce conductivity. However, the model also suggests that an increase in silicon roundness enhances conductivity, a finding that directly contradicts established physical principles. This discrepancy is likely attributable to multicollinearity among the statistical parameters, which can introduce significant bias into the partial regression coefficients. Consequently, the validity of each coefficient must be individually verified. Prior to the *t*-test, the normality of residuals was verified using the Shapiro–Wilk test (*p* > 0.05), confirming the validity of the parametric assumption. In this study, a *t*-test was employed to validate the significance of each partial regression coefficient, using the following formula:

(4) $$t_{j}\;=\;\frac{\beta_{j}}{S_{\beta_{j}}},$$

where $\beta_{j}$ represents the estimated value of the partial regression coefficient, and $S_{\beta_{j}}$ denotes the standard error of $\beta_{j}$.

The hypotheses for the *t*-test are established as follows: The null hypothesis H_0_ states that the partial regression coefficient $\beta_{j}$ equals zero, implying no significant linear relationship between the j-th independent variable and the electrical conductivity; the alternative hypothesis H_1_ asserts the opposite $H_{0}:\beta_{j}\;=\;$0; $\;H_{1}:\beta_{j}\;\neq \;0$.

The significance level (α) for the test is set at 0.05.

If |$t_{j}$|≥$\;t_{\frac{\alpha }{2},n-m-1}$, the null hypothesis $H_{0}$ is rejected in favor of the alternative hypothesis ($H_{1}$), demonstrating a significant linear relationship between the given independent variable and the dependent variable.

Table 2 summarizes the partial regression coefficients and their corresponding *t*-values. According to the t-distribution critical value table, the threshold is t0.05/2.17 = 2.11. The analysis reveals that |t6| > |t2| > 2.11, with *p*-values less than 0.005. This indicates that variables X2 and X6 successfully pass the significance test and are statistically significant. Conversely, the remaining independent variables fail to meet this criterion and are therefore deemed statistically insignificant.

**Figure 5:**
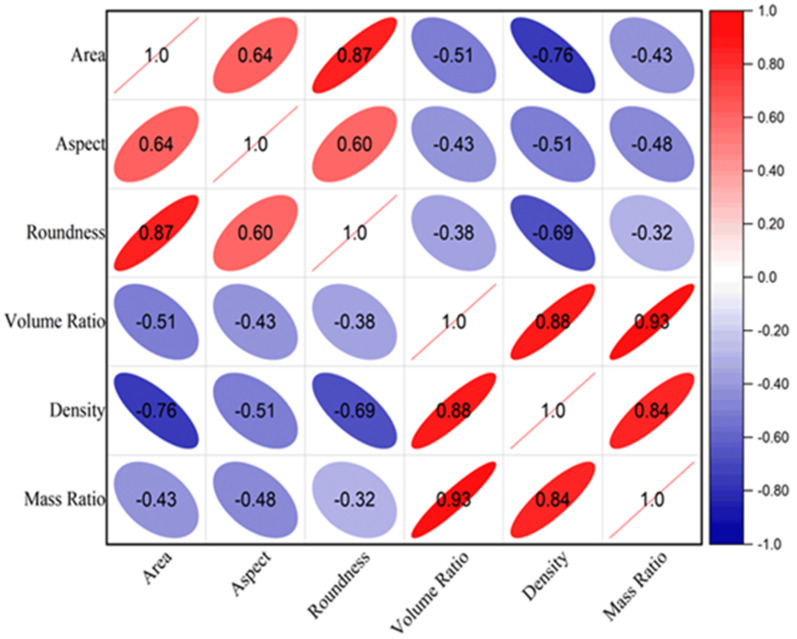
Correlation heat map of eutectic silicon statistical indicators.

Further Pearson correlation analysis reveals a high degree of multicollinearity among the statistical parameters, as illustrated in Figure 5. In the correlation matrix, red and blue regions denote positive and negative correlations, respectively. The correlation coefficients range from −1 to 1, with their absolute values indicating the strength of the linear relationship. Notably, the positive correlation coefficient between the area and roundness of the eutectic silicon reaches 0.87, while the coefficient between volume fraction and surface density is as high as 0.88. Consequently, ordinary multiple linear regression fails to decouple the interactive effects among these parameters, ultimately leading to distortion of the regression model.

### 3.2. Construction of the Regression Model Based on Principal Component Regression (PCR)

**Figure 6:**
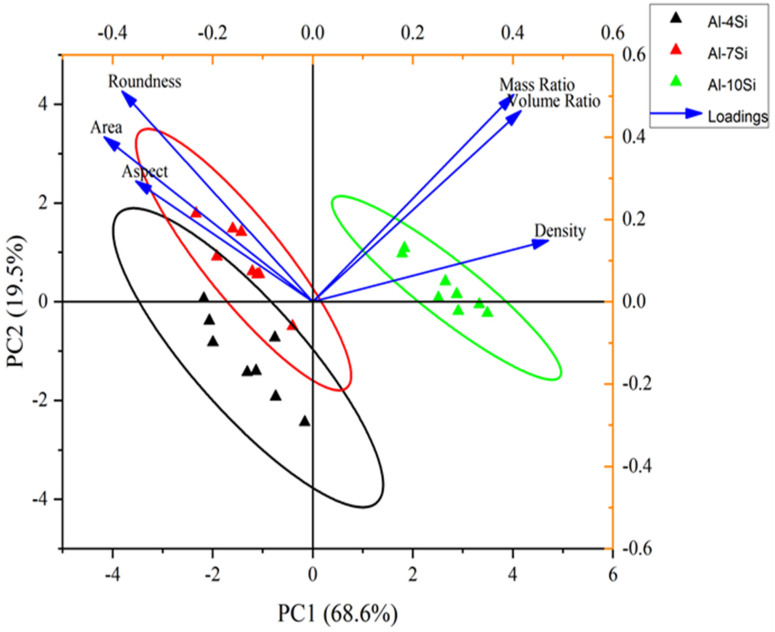
Score distribution chart of each statistical point.

To overcome the limitations imposed by multicollinearity, principal component regression (PCR) was employed to achieve dimensionality reduction. Through SIMCA 18 software, two principal components (PC1 and PC2) were extracted, yielding a cumulative variance contribution rate of 88.1%. This high percentage demonstrates that these two components successfully capture the vast majority of the information inherent in the original dataset. As illustrated by the scatter plot of the sample data in Figure 6, the six statistical parameters can be distinctly clustered into two primary categories. Specifically, PC1 predominantly couples the spatial distribution and overall content of the eutectic silicon (i.e., surface density, volume fraction, and mass fraction). Conversely, PC2 intrinsically characterizes its morphological features (i.e., area, aspect ratio, and roundness). Based on the principal component score matrix, the mathematical formulations for PC1 and PC2 can be deduced as follows:

(5) $$\mathrm{PC}1\;=\;-0.42X_{1}\;-\;0.35X_{2}\;-\;0.38X_{3}+\;0.42X_{4}+\;0.47X_{5}\;+\;0.4X_{6}$$

(6) $$\mathrm{PC}2=0.4X_{1}+0.29X_{2}+0.51X_{3}+0.46X_{4}+0.15X_{5}+0.5X_{6}$$

Based on the mathematical formulations of PC1 and PC2, the composite scores for each sample across the two principal components were calculated. Subsequently, a multiple linear regression was executed by assigning these composite scores as the independent variables and the electrical conductivity (Y) as the dependent variable, yielding:

(7) Y= −1.33PC1 − 1.61PC2 + 37.48

A *t*-test was conducted on the regression model, utilizing a critical *t*-value of 2.08, with the results summarized in Table 3. The analysis demonstrates that both independent variables in the fitted equation derived via the PCR method successfully passed the *t*-test, thereby confirming their statistical significance. By substituting the mathematical formulations of PC1 and PC2 back into the model, the final regression equation can be expressed as:

(8) $$Y=\;-0.0854X_{1}\;-\;0.001{4X}_{2}-\;0.31{57X}_{3}\;-1.2992X_{4}-\;0.8666X_{5}-1.33{01X}_{6}\;+\;37.48$$

The statistical results reveal that in as-cast eutectic Al-Si alloys, PC1 (accounting for 68.6% of the variance)—which represents the silicon-content parameters—exerts the most profound impact on electrical conductivity. Specifically, the volume fraction exhibits the strongest suppressive effect. Furthermore, within alloys of identical chemical composition, the size and roundness of individual eutectic silicon particles play a decisive role in dictating the fluctuations in conductivity.

## 4. Discussion

### 4.1. Construction of a Semi-Quantitative Electron Drift Path Model Based on PCR Factors

Electrical conductivity measurements of the three alloys under varying conditions reveal that conductivity decreases with increasing Si content (e.g., the conductivity of as-cast Al-4Si is 39–45% IACS, whereas that of Al-10Si drops to 32–34% IACS). The prevailing consensus postulates that the lattice-distortion layer at the Al/Si interface induces intense electron scattering [23,24,25]. However, this theory encounters a contradiction when applied to microstructural gradients induced by varying cooling rates [26,27]: as the cooling rate accelerates, the silicon particles become smaller and more densely distributed, which inherently leads to a proliferation of phase interfaces and a thickening of the lattice distortion layer. Theoretically, this enhanced scattering should result in a deterioration of electrical conductivity. In stark contrast, the experimental results herein demonstrate that the refinement of silicon particles induced by rapid solidification actually enhances the electrical conductivity. Thus, the theoretical prediction completely contradicts the empirical observations. According to Matthiessen’s rule, electrical resistivity depends on electron scattering at intrinsic lattices and microstructural defects, including phase boundaries [28,29]. The refinement of eutectic silicon inevitably increases the density of Al/Si interfaces. These interfaces act as dense scattering centers that truncate the electron mean free path (MFP), consequently reducing macroscopic conductivity [30]. Additionally, deviations from a spherical morphology enlarge the electron scattering cross-section of secondary phases, which further amplifies the resistive penalty [16].

**Figure 7:**
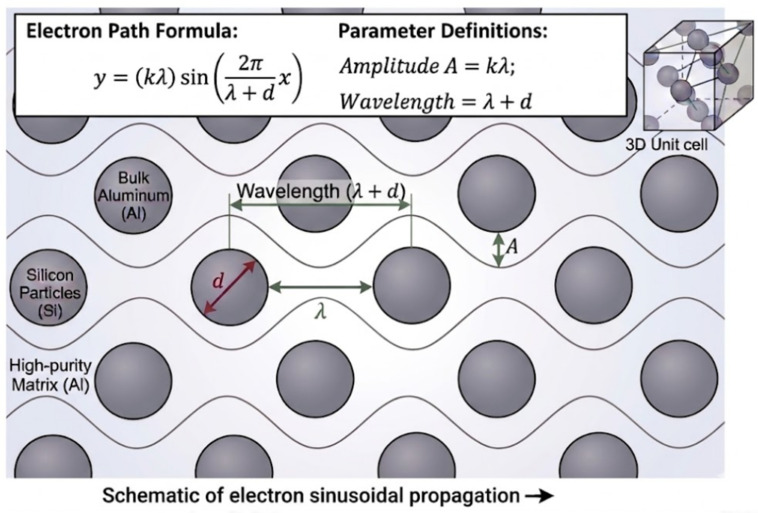
Geometric model of sine-wave electron drift path.

To rigorously elucidate this anomalous phenomenon and substantiate the factor weights extracted via the aforementioned PCR analysis, this study proposes a microscopic “semi-quantitative model for electron drift paths.” Within this physical framework, the eutectic silicon is treated as a semiconducting phase that does not contribute to electrical conduction. Instead, driven by an electric field, itinerant electrons will drift along the path of least resistance, bypassing the eutectic silicon entirely within the continuous α-Al matrix, as schematically illustrated in Figure 7.

Within this mechanistic framework, the modulatory effects of the two principal components (PC1 and PC2) derived from the PCA regression on electrical conductivity acquire explicit physical validation:

(1) PC1 (Content) corresponds to the truncation effect of scattering interfaces: As the directional migration of electrons along the applied current vector is obstructed by eutectic silicon, a higher volume fraction of eutectic silicon (PC1) inherently increases the frequency at which electrons encounter insulating barriers per unit path length. This fundamentally truncates the electron “mean free path” (MFP) within the matrix, thereby acting as the primary factor suppressing macroscopic electrical conductivity.
(2) PC2 (Morphology) corresponds to the geometric tortuosity of the drift paths: To precisely calculate the resistive penalty imposed by the silicon particle morphology on the electron bypass trajectories, a sine-wave drift path model is established in this study. Assuming that the silicon particles are completely spheroidized and periodically arrayed within the eutectic region, the electron drift trajectory can be mathematically fitted to a sine-wave function:

(9) $$y=A\sin\frac{2\pi }{L_{0}}x,$$

where L0 = λ + d, denotes the horizontal spatial period required for an electron to complete a single bypass cycle (λ being the inter-particle spacing and d the equivalent particle diameter). To estimate the geometric deviation of the electron path, a simplified analysis based on a regular array structure was conducted in this study. It should be noted that the actual electron transport process does not follow a single deterministic path, but is rather dictated by the distribution of the electric potential field. Therefore, the sinusoidal path introduced herein serves solely as an equivalent geometric approximation. Its amplitude should not be simply determined by the geometric center position; rather, it is governed by the effective width of the conductive channel (i.e., the inter-particle spacing λ), yielding an amplitude of A = kλ (k ≈ 0.2~0.5).

To calculate the actual drift distance L traversed by the electrons, an arc length integration is performed on the trajectory equation:

(10) $$\frac{\mathrm{dy}}{\mathrm{dx}}=\frac{2\pi A}{L_{0}}\times \cos\frac{2\pi }{L_{0}}x,$$

Substituting the derivative into the arc length integral formula yields the actual distance L traversed by an electron within a single period:

(11) $$L=\int_{0}^{T}\sqrt{1+{\left(\frac{\mathrm{dy}}{\mathrm{dx}}\right)}^{2}}=\int_{0}^{T}\sqrt{1+{\left(\frac{2\pi A}{L_{0}}\right)}^{2}\times \cos^{2}(\frac{2\pi }{L_{0}}x)}\mathrm{dx},$$

This integral represents a classic complete elliptic integral of the second kind. By applying a Taylor series expansion for approximation, the expression for the single-period path length can be obtained:

(12) $$\;L\;\approx {\;L}_{0}+\frac{\pi^{2}A^{2}}{L_{0}},$$

From Equation (12), it is evident that the increment in the actual electron path (which represents the additional resistive penalty) is directly proportional to the square of the detour amplitude A.

By further simplifying Equation (12), the expression for path tortuosity τ (defined as the actual path length per unit of linear distance) can be derived as follows:

(13) $$\tau =\frac{L}{L_{0}}\;\approx \;1\;+\frac{\pi^{2}A^{2}}{{L_{0}}^{2}}=1\;+\;\pi^{2}\times {\left(\frac{k}{1+\frac{d}{\lambda }}\right)}^{2},$$

Equation (13) reveals a fundamental physical mechanism: the increment in the actual electron path length (namely, the microscopic path tortuosity L/L0) is predominantly governed by the square of the morphology-induced amplitude-to-linear-distance ratio (A/L0). Consequently, when the eutectic silicon deviates from a spherical morphology and adopts an elongated lath-like or plate-like structure (i.e., a pronounced increase in aspect ratio), the A/L0 ratio escalates accordingly. This geometric transition triggers a non-linear prolongation of the electron drift path, which in turn drastically amplifies the macroscopic electrical resistance.

### 4.2. Non-Linear Amplification Effect of Eutectic Silicon Morphological Evolution on Macroscopic Electrical Resistance

To accurately calculate the electron drift path length using the proposed model, a representative domain of 1000 × 1000 μm^2^ is assumed, within which the area fraction of the eutectic silicon particles is set to 50%. The calculated results are summarized in Table 4. Within this table, the terms “parallel” and “perpendicular” denote the morphological elongation of the particles aligned with and orthogonal to the applied current direction, respectively; the variable d represents the equivalent diameter of the eutectic silicon particles; and the “arc length/period” ratio characterizes the “tortuosity” of the electron drift trajectory relative to the direct linear distance.

Based on the geometric calculations presented in Table 4, the path tortuosity (L/L0) for ideal spherical particles exhibits a pronounced size-insensitivity. As the equivalent diameter d increases from 3 μm to 9 μm, the amplitude of an individual bypass trajectory inherently increases; however, because the spatial period T (or L0) scales proportionally, the L/L0 ratio remains practically constant at approximately 1.18. In stark contrast, morphological deviations from sphericity (i.e., an increased aspect ratio) exert a highly asymmetric influence on the electron drift trajectories. As evidenced by Table 4, if the lath-like eutectic silicon aligns perfectly parallel to the current vector, the resultant L/L0 ratio (1.01 at an aspect ratio of 4) is actually marginally lower than that of an ideal spherical particle. However, in actual as-cast microstructures solidified under non-directional conditions, coarse lath-like eutectic silicon particles are randomly interlocked and interspersed throughout the three-dimensional space. This inherent randomness dictates the inevitable presence of numerous plate-like silicon structures oriented perpendicular to, or intersecting at high angles with, the applied current vector. Geometric calculations confirm that the path tortuosity penalty imposed by perpendicular orientations (where the L/L0 ratio escalates to 1.80, representing an increase of approximately 52.5%) vastly outweighs the marginal geometric gain afforded by parallel orientations (a reduction of merely 14.4%) in absolute magnitude.

**Figure 8:**
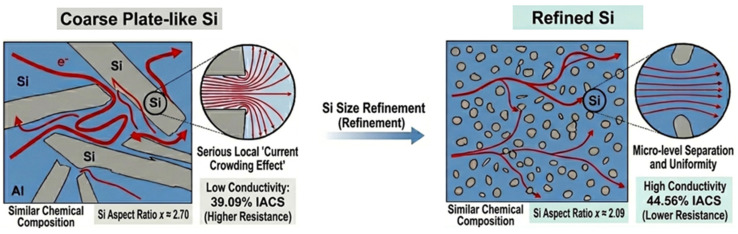
Explanation of Si Refinement on 3D Conductive Network Optimization and Macro-Resistance Reduction.

As illustrated in Figure 8, within the complex macroscopic three-dimensional conductive network, these coarse laths oriented at high angles to the applied current vector constitute rate-limiting “bottlenecks”. They not only compel a massive influx of electrons to navigate highly tortuous bypass trajectories but also induce a severe localized “current crowding effect” due to their extensive truncation of the continuous matrix. Consequently, this non-linear geometric prolongation statistically dominates the escalation in the system’s equivalent electrical resistance, directly governing the upper limit of the alloy’s macroscopic electrical conductivity. Using the Al-4Si alloy from Table 1 as an illustrative case: under comparable chemical compositions, the sample with a significantly optimized morphology (aspect ratio X2 = 2.09) exhibits a superior electrical conductivity of 44.56% IACS. In contrast, the sample characterized by coarse, pronounced lath-like structures (aspect ratio X2 = 2.70) yields a conductivity of merely 39.09% IACS. The fundamental origin of this performance discrepancy of approximately 5% IACS lies precisely in the path tortuosity induced by the elongated silicon particles, which drastically amplifies the macroscopic electrical resistance.

### 4.3. Optimization of the Three-Dimensional Conductive Network via Microstructural Refinement

Importantly, while a mere reduction in absolute size does not alter the relative tortuosity of an individual bypass trajectory, the microstructural refinement induced by rapid solidification yields a pronounced statistical enhancement in overall electrical conductivity. For a constant silicon volume fraction, the refinement of eutectic silicon inherently dictates a dramatic proliferation in the number surface density of particles per unit volume. From a statistical physics perspective, this microstructural evolution engenders two critical effects:

Firstly, densification and homogenization of conductive channels: As elucidated by the microscopic isolation and homogenization model in the right panel of Figure 8 (Refined Si), although the inter-particle spacing λ narrows correspondingly, the proportional reduction in particle diameter d facilitates a geometric multiplication in the number of effective conductive channels (i.e.,α-Al interstices) within the matrix. This high-density distribution of channels ensures a significantly more uniform current distribution at the microscale. Consequently, it effectively neutralizes the severe localized “current crowding effect” generated at the peripheries of coarse phases, as depicted in the left panel of Figure 8 (Coarse Plate-like Si);

Secondly, enhancement of matrix connectivity: The partitioning effect of fine silicon particles on the α-Al matrix behaves more as a “dispersion” rather than a rigid “truncation”. Compared to the continuous barrier walls typically formed by coarse plate-like silicon, the refined eutectic microstructure guarantees a high degree of three-dimensional connectivity within the aluminum matrix, thereby providing itinerant electrons with a multiplicity of alternative bypass trajectories [31].

In summary, the refinement and spheroidization of eutectic silicon confer a synergistic dual benefit: refinement optimizes the quantitative distribution of conductive channels to remarkably diminish the system’s equivalent macroscopic resistance, whereas spheroidization effectively mitigates electron scattering losses by minimizing geometric path tortuosity.

## 5. Conclusions

In this study, a Principal Component Regression (PCR) model was coupled with a microscopic electron transport model to quantitatively analyze the effects of eutectic silicon evolution on the electrical conductivity of Al-Si alloys. The principal conclusions are drawn as follows:

- A Principal Component Regression (PCR) model was established to quantitatively characterize the “microstructure-property” relationship in Al-Si alloys, effectively eliminating the distortion caused by the multicollinearity of eutectic silicon parameters;
- The “content factor” (PC1, variance contribution of 68.6%), which is dominated by volume fraction, plays a major role in determining the electrical conductivity. Meanwhile, under a constant chemical composition, the “morphological factor” (PC2, 19.5%), centered on roundness, exerts a precise modulatory effect;
- High-density eutectic silicon increases baseline resistance by truncating the electron mean free path (MFP). Additionally, elongated silicon particles (high aspect ratio) non-linearly extend the electron drift paths, significantly degrading electrical conductivity;
- The refinement and spheroidization of eutectic silicon provide a dual benefit: the former optimizes the quantitative distribution of conductive channels, whereas the latter effectively mitigates electron scattering losses by minimizing geometric path tortuosity, demonstrating a clear predictive advantage over empirical testing and providing actionable criteria for designing lightweight, high-conductivity Al-Si castings used in new energy vehicle power systems.

## 6. Outlook

Future investigations will incorporate high-resolution X-ray tomography to directly map the spatial tortuosity of the continuous eutectic network, thereby upgrading current stereological approximations to actual 3D conductive topologies. Additionally, the proposed electron drift model will be expanded to account for thermal lattice vibrations and concurrent mechanical stress, simulating the authentic high-temperature operating environments of electric vehicle powertrains.

## Figures and Tables

**Table 1:** Statistical results of conductivity, morphology, distribution and content of eutectic silicon.

Alloy	Electrical Conductivity %IACS	Area μm^2^	Aspect Ratio	Roundness	Volume Fraction Vol.%	Surface Density 1/μm^2^	Mass Fraction wt.%
	Y	X_1_	X_2_	X_3_	X_4_	X_5_	X_6_
Al-4Si	40.49	2.22	2.44	2.33	7	0.03	4
	41.08	2.38	2.20	2.23	6	0.02	4
	39.68	3.43	2.78	2.55	10	0.03	4
	40.43	2.85	2.39	2.36	13	0.05	4
	41.72	2.44	2.52	2.22	6	0.02	4
	44.56	2.07	2.09	2.04	7	0.02	4
	39.60	2.88	2.70	2.37	6	0.02	4
	39.09	3.39	2.70	2.42	9	0.02	4
Al-7Si	38.55	3.55	2.40	2.71	12	0.04	7
	38.06	3.95	2.60	2.89	16	0.03	7
	39.35	3.24	2.20	2.36	11	0.02	7
	37.47	4.51	2.55	2.68	18	0.02	7
	38.63	4.53	2.42	2.48	13	0.04	7
	38.57	3.46	2.35	2.79	12	0.03	7
	37.98	4.60	2.52	2.71	10	0.03	7
	37.71	4.53	2.69	3.00	13	0.03	7
Al-10Si	34.35	1.93	2.26	2.51	24	0.13	10
	32.94	1.53	2.30	1.99	21	0.17	10
	33.16	1.55	2.15	2.05	24	0.16	10
	33.83	1.34	2.11	1.97	23	0.14	10
	34.48	1.88	2.34	2.38	24	0.13	10
	32.88	1.81	2.26	2.05	24	0.18	10
	32.08	1.13	2.24	1.75	24	0.21	10
	32.90	1.12	2.35	1.75	24	0.21	10

**Table 2:** Partial regression coefficient values and *t*-values.

Variable	Partial Regression Coefficient	Standard Error	*t*-Value
X1	−0.049	0.39	−0.13
X2	−4.54	1.52	−2.99
X3	0.35	1.37	0.25
X4	−3.37	8.34	−0.40
X5	−21.34	13.31	−1.60
X6	−83.57	26.02	−3.21
Intercept	55.61	3.07	18.11

**Table 3:** Statistical table of fitting results.

Variable	Partial Regression Coefficient	*t*-Value	Critical *t*-Value
PC1	−1.33	−12.13	2.08
PC2	−1.61	−7.81	
Intercept	37.48	171.99	

**Table 4:** Calculation of the length of the electron drift path.

Eutectic Si Morphology	d	λ (μm)	Number of Rows	Amplitude	Period	Arc Length/Period
Spherical	3	1.04	248	0.58	4.04	1.18
Spherical	6	2.08	124	1.17	8.08	1.18
Spherical	9	3.12	83	1.75	12.12	1.18
Aspect ratio = 2 (Parallel)	3	---	248	0.41	5.99	1.05
Aspect ratio = 2 (Perpendicular)	3	---	248	0.82	4.52	1.32
Aspect ratio = 4 (Parallel)	3	---	248	0.29	8.18	1.01
Aspect ratio = 4 (Perpendicular)	3	---	248	1.17	5.22	1.80

## Data Availability

The original contributions presented in this study are included in the article. Further inquiries can be directed to the corresponding author.

## Nomenclature

**Abbreviations**	**Description/Definition**
SEM	Scanning Electron Microscopy
PCR	Principal Component Regression
NEVs	New Energy Vehicles
MFP	Mean Free Path
MLR	Multiple Linear Regression
SE	Secondary Electron
IACS	International Annealed Copper Standard
**Symbols**	**Description/Definition**
α-Al	Primary aluminum matrix phase
S	Roundness of an individual particle (Equation (1))
A	Area of an individual particle (Equation (1))
P	Perimeter of an individual eutectic silicon particle
X_1_	Average area of eutectic silicon
X_2_	Aspect ratio of eutectic silicon
X_3_	Roundness of eutectic silicon
X_4_	Volume fraction of eutectic silicon
X_5_	Surface Density of eutectic silicon
X_6_	Mass fraction of added silicon
Y	Electrical conductivity (Dependent variable)
β_0_	Regression constant (intercept)
β_j_	Partial regression coefficient
ε	Residual error term
t_j_	*t*-value for significance test
S_βj_	Standard error of partial regression coefficient
PC1	First Principal Component (Content factor)
PC2	Second Principal Component (Morphological factor)
L_0_	Horizontal spatial period of electron drift
λ	Inter-particle spacing of eutectic silicon
d	Equivalent particle diameter of eutectic silicon
k	Proportionality constant for drift amplitude
L	Actual drift distance traversed by an electron
τ	Path tortuosity (L/L0)
